# Clinically applicable parasite viability assay for rapid assessment of antimalarial pharmacodynamic endpoints

**DOI:** 10.1128/aac.01863-24

**Published:** 2025-06-17

**Authors:** Mohamed Maiga, Sebastian G. Wicha, Fatoumata Diallo, Issa Traoré, Abdoul Karim Samaké, Fanta Sogore, Ousmaila Diakité, François Dao, Djeneba Diallo, Aliou Traoré, Abdoulaye A. Djimde, Thomas Spangenberg, Claudia Demarta-Gatsi, Laurent Dembele

**Affiliations:** 1 Faculty of Pharmacy, Malaria Research and Training Centre (MRTC), Université des Sciences, des Techniques et des Technologies de Bamako (USTTB) 225803, Bamako, Mali; 2 Department of Clinical Pharmacy, Institute of Pharmacy 276709, University of Hamburg, Hamburg, Germany; 3 Global Health R&D of Merck Healthcare, Ares Trading S.A. (an affiliate of Merck KGaA, Darmstadt, Germany), Eysins, Switzerland; The Children's Hospital of Philadelphia, Philadelphia, Pennsylvania, USA

**Keywords:** *Plasmodium*; parasite viability, MitoTracker, parasite reduction ratio, pharmacodynamics

## Abstract

Evaluating the efficacy of antimalarial drugs is crucial in the fight against malaria, as the parasiticidal effectiveness of these drugs often predicts their clinical success. Parasite Reduction Ratio (PRR) assay is the current method of choice for assessing antimalarial’s ability to halt parasite recovery after treatment; however, it is time-consuming and resource-intensive, making it less ideal for low-resource or clinical settings. Recent advancements in parasite viability assessment, such as use of the MitoTracker (MT) which probes stain active mitochondria in live cells, provide a faster way to distinguish live from dead parasites using the flow cytometry, providing, thus, timely insights to inform treatment outcomes in clinical trials. In this study, the accuracy of direct viability assessment (DVA) of the parasite using MT staining was compared with the previously established PRR assay to evaluate the efficacy of four reference antimalarial drugs (dihydroartemisinin, chloroquine, atovaquone, and pyrimethamine) using *P. falciparum* 3D7 strain. Additionally, a mathematical model was developed to estimate key parameters, such as maximum killing rate and lag phase. The model yielded comparable values for these compounds across both assays reinforcing the reliability of the DVA assay for rapidly assessing antimalarial drug efficacy. In conclusion, the DVA relies on specialized equipment and technical expertise. However, it can emerge as an alternative to the PRR, offering a faster and more clinically suited approach for studies.

## INTRODUCTION

Assessing the effectiveness of drugs against parasites is pivotal in combating parasitic diseases, particularly malaria, which despite concerted efforts, persist as a significant global health challenge (1). Effective control strategies depend on the ability of antimalarial drugs to kill parasites (parasiticidal efficacy) and, therefore, constitute a surrogate predictor for clinical efficacy (2). However, assessing parasite viability directly based on patient survival is complex, time-consuming, and costly. Instead, the presence of circulating parasites is used as a surrogate, but this method does not distinguish viable and dead parasites accurately and relies on the skills of the microscopy reader. Therefore, it is crucial to assess parasite viability after treatment and distinguish it from parasite clearance—the elimination of detectable parasites from the host—to guide therapeutic interventions and address the growing concern of drug resistance in malaria-endemic regions (3–5). This distinction is nevertheless important as parasite clearance rate is composed of two key components: (i) the drug’s speed of action and (ii) the host ability to remove circulating parasites (2, 6). Determining viability would essentially allow clinicians to discriminate fast-killing followed by intermediate or slow parasite clearance from slow-killing with slow clearing profiles (7).

In non-clinical studies, the *in vitro* parasite reduction ratio (PRR) (3, 8) assay serves as the standard method for evaluating antimalarial drug efficacy by assessing the ability of a single parasite to re-establish a population after drug removal. Through limiting dilution and subsequent parasite regrowth, the assay evaluates the drug’s impact on parasite viability and growth dynamics by quantifying its killing rate and speed of action over time under controlled *in vitro* conditions. The resulting viability-time profile enables the determination of key pharmacodynamic (PD) parameters, such as lag phase, and maximum killing rate (*E*_max_). Thus, while the PRR assay is valuable for screening drug candidates using *Plasmodium* laboratory strains (i.e., *P. falciparum* 3D7 and NF54), its application in field settings encounters significant challenges (9). Typically, *P. falciparum* field isolates can be cultured for a maximum of 72–96 h, whereas the PRR assay necessitates 14–21 days of continuous culture, which presents a substantial time constraint. Compounding this issue is the variability in parasite growth rates—reflecting the parasites’ adaptation to *in vitro* culture—and the increased risk of contamination during prolonged culture periods. Additionally, the assay is resource-intensive, requiring specialized laboratory facilities and technical expertise that may not be readily available in field settings. These limitations preclude a rapid assessment of the parasiticidal efficacy of antimalarial drugs in clinical trials. This highlights the need for an alternative method that delivers rapid and reliable results to assess treatment outcomes and advance the clinical development of antimalarial drugs.

The assessment of cell viability using fluorescent dyes has emerged over time and can provide crucial insights into the functional state of the parasites, distinguishing between viable, dead, and damaged parasites (10). The use of MitoTracker (MT) staining, a dye that selectively accumulates in metabolically active mitochondria of viable parasites, offers several distinct advantages. First, it enables direct visualization of viable parasites (11) by flow cytometry and/or complementary analytical techniques, enhancing accuracy compared to traditional techniques (e.g., Microscopy or PCR). Second, it allows for direct viability assessment (DVA) of the parasite, making it suitable for use in field settings. Moreover, by differentiating between viable and non-viable parasites, this method offers valuable insights into the effect of drugs on parasite survival (12), thus improving the accuracy of clinical trial outcomes and informing treatment protocol decisions. Furthermore, the combination of parasite DVA (MT staining) and parasite growth monitoring (SYBR staining) offers a more comprehensive picture of infection progression as well as drug parasiticidal efficacy (13).

**Fig 1:**
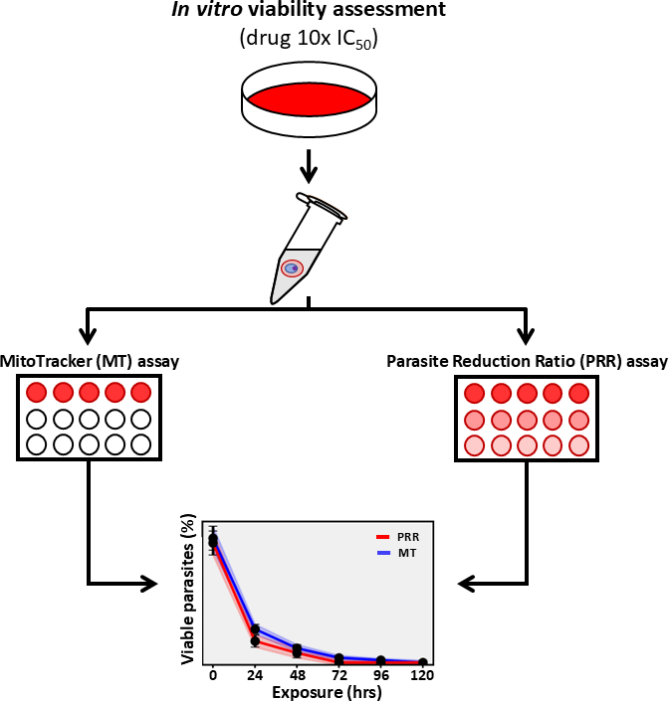
Experimental setups for *in vitro* PRR and DVA assays: a comparative analysis. *P. falciparum* 3D7 strain was exposed to antimalarials (10 × IC_50_). Samples were collected at 0, 24, 48, 72, 96, and 120 h, then washed and distributed into 96-well plates—each compound was tested in triplicate, with eight replicates for the 0 h time point. For the DVA assay, plates were immediately stained with MT Deep Red FM, incubated for 1 h, and analyzed via flow cytometry. While for the PRR assay, each sample underwent fourfold serial dilutions, followed by a 14-day incubation. Parasite viability was determined through microscopy and flow cytometry based on the dilution limit.

In this study, we compared the efficacy of the traditional PRR assay with the innovative DVA assay using MT staining for assessing the speed of action of antimalarial drugs (Fig. 1). Through the validation and subsequent application of the DVA assay in clinical evaluations, we could provide timely insights into the viability of *Plasmodium* parasites after drug treatment.

## RESULTS

### Experimental results

**Fig 2:**
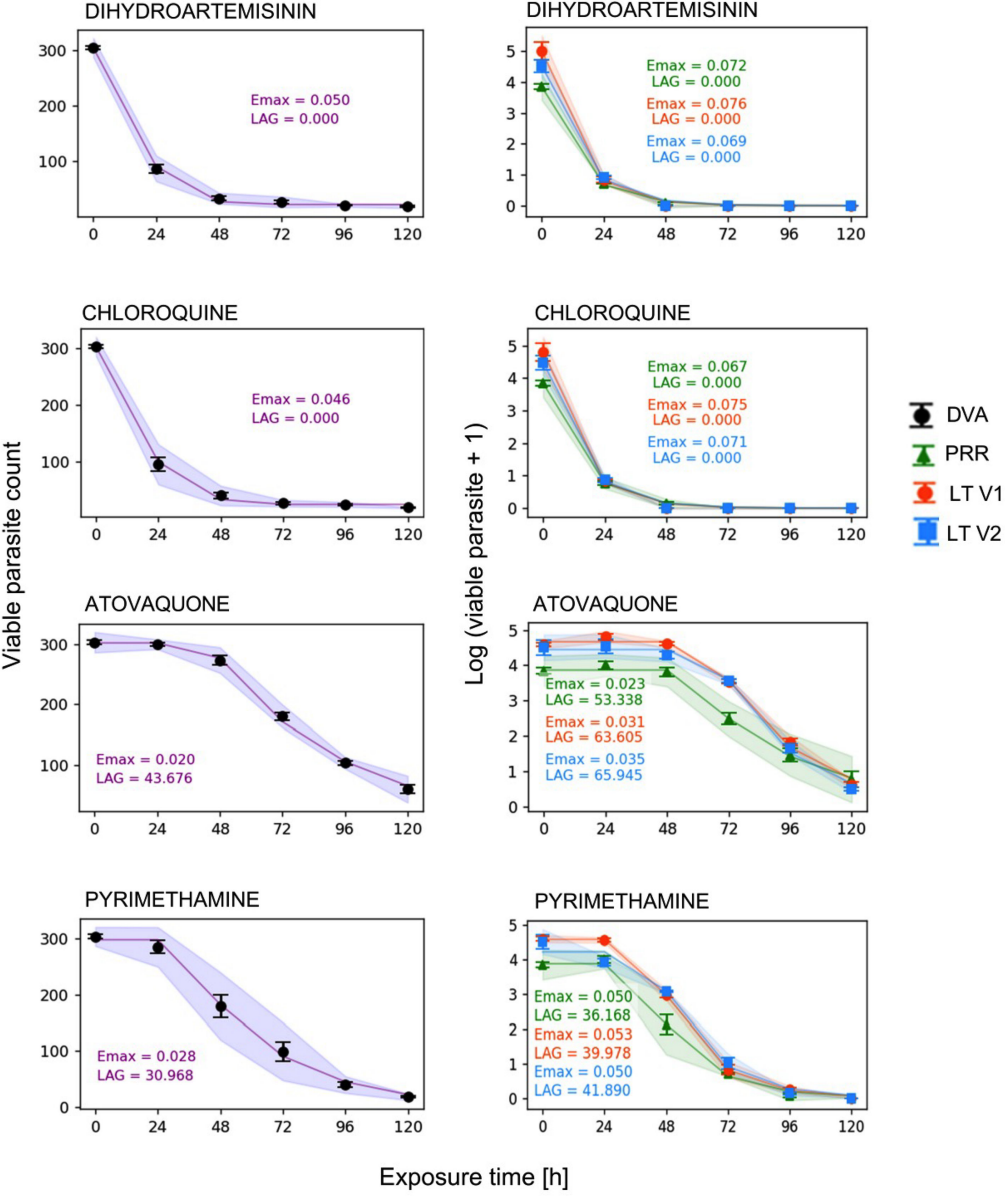
Comparison of the killing patterns of the experimental DVA assay versus the experimental and literature (LT) PRR assays. The dots represent the observation data, while the line represents the model prediction. The maximum killing effect (*E*_max_) and lag phase (LAG) are estimated parameters (Table 1). DVA, direct viability assessment assay; PRR, experimental parasite reduction ratio assay data; LT V1, parasite reduction ratio assay data from literature (8); LT V2, parasite reduction ratio assay data from literature (3); Error bars represent SEM (three independent experiments, each done in triplicate). The shaded area indicates the standard deviation.

To compare the two assays, we overlaid the DVA and PRR results from simultaneously conducted experiments (Fig. 1). Additionally, to enhance the comparison, we considered data from the literature (LT) on the PRR, specifically the initial standardized version of the PRR assay by Sanz et al. (PRR version 1, V1) (8) and the more recent, streamlined version by Walz et al. (PRR version 2, V2) (3), focusing on dihydroartemisinin (DHA), chloroquine (CQ), atovaquone (ATO), and pyrimethamine (PYRI). The results show that the DVA assay aligns with the killing pattern of the PRR assay (Fig. 2, PRR).

### Model-based comparison suggests alignment of DVA assay with PRR assay

For the PRR, we used percentage viable parasites, whereas absolute viable parasite count from flow cytometry was used for the DVA. Hence, a mathematical model was developed to characterize and compare the killing kinetics. Specifically, we estimated the PD parameters maximum killing rate (*E*_max_), which describes the maximum possible exponential decline of parasites under excess drug exposure (i.e., 10 × IC_50_), and the lag phase, which captures the time interval until killing started after drug exposure.

The obtained killing profiles for the DVA and PRR assays are presented in Fig. 2. DHA and CQ exposure led to a rapid and marked decreases in the numbers of viable parasites within the first 24 h. By 48 h, parasite levels had dropped to a critical minimum, remaining stable at extremely low levels up to 120 h for both DHA and CQ. *E*_max_ values of 0.050 h^−1^ in the DVA assay and 0.070 h^−1^ in the PRR assay were quantified indicating rapid killing. The absence of a lag phase indicates that the PD effects were immediate in both assays with no apparent time delay upon drug administration.

ATO exposure demonstrated slower killing kinetics, with consistent killing profiles observed across both assays. The lag phase was 44 h ± 1.18 in the DVA assay and 53 h ± 4.7 in the PRR assay, reflecting the delayed onset of ATO’s action. *E*_max_ was quantified to 0.02 h^−1^ for the DVA and 0.023 h^−1^ for the PRR assay indicating a slower killing rate compared to the other studied antimalarials.

In the case of PYRI, a gradual reduction in viable parasite numbers was observed with both evaluation methods. The PRR assay showed an *E*_max_ of 0.05 h^−1^ and a lag phase of 36 h, while the DVA assay reported an *E*_max_ of 0.03 h^−1^ and a lag phase of 31 h. These results indicate that PYRI exhibits an intermediate effect, falling between the rapid killing profiles of DHA and CQ and the slower action of ATO. All estimated parameters are presented in Table 1.

**TABLE 1:** Summary table of PD parameters estimates from the final model^*b*^^,^^*c*^

Drug	Parameters	Estimate (CI_95%_)			
		DVA (experimental)	PRR (experimental)	PRR LT V1 (Sanz et al.)	PRR LT V2 (Walz et al.)
Artemisinin	*E*_max_ [h^−1^]	0.050 [0.046–0.053]	0.072 [0.060–0.079]	0.076 [0.065–0.086]	0.070 [0.063–0.076]
	Lag phase [h]	0^*a*^	0^*a*^	0^*a*^	0^*a*^
Chloroquine	*E*_max_ [h^−1^]	0.046 [0.045–0.054]	0.067 [0.052–0.067]	0.075 [0.066–0.083]	0.071 [0.062–0.079]
	Lag phase [h]	0^*a*^	0^*a*^	0^*a*^	0^*a*^
Atovaquone	*E*_max_ [h^−1^]	0.02 [0.001–0.021]	0.023 [0.014–0.025]	0.031 [0.027–0.034]	0.035 [0.029–0.040]
	Lag phase [h]	43.7 [41.4–46.6]	53.3 [44.1–63.0]	63.6 [61.1–66.1]	65.9 [63.3–68.5]
Pyrimethamine	*E*_max_ [h^−1^]	0.028 [0.022–0.037]	0.050 [0.029–0.070]	0.053 [0.047–0.058]	0.051 [0.040–0.061]
	Lag phase [h]	31.0 [24.3–37.7]	36.2 [30.6–43.8]	40.0 [38.4–41.6]	41.9 [39.4–44.4]

^*a*^ Fixed to final estimate.

^*b*^ CI95%: 95% confidence intervals of the estimate.

^*c*^ DVA, direct viability assessment; PRR, experimental parasite reduction ratio assay data; LT, literature; V1, parasite reduction ratio assay data from literature (9); V2, parasite reduction ratio assay data from literature (3).

### The DVA assay demonstrates higher maximum effects and shorter lag phases compared to PRR assay, while maintaining a strong quantitative and qualitative correlation between the methods

**Fig 3:**
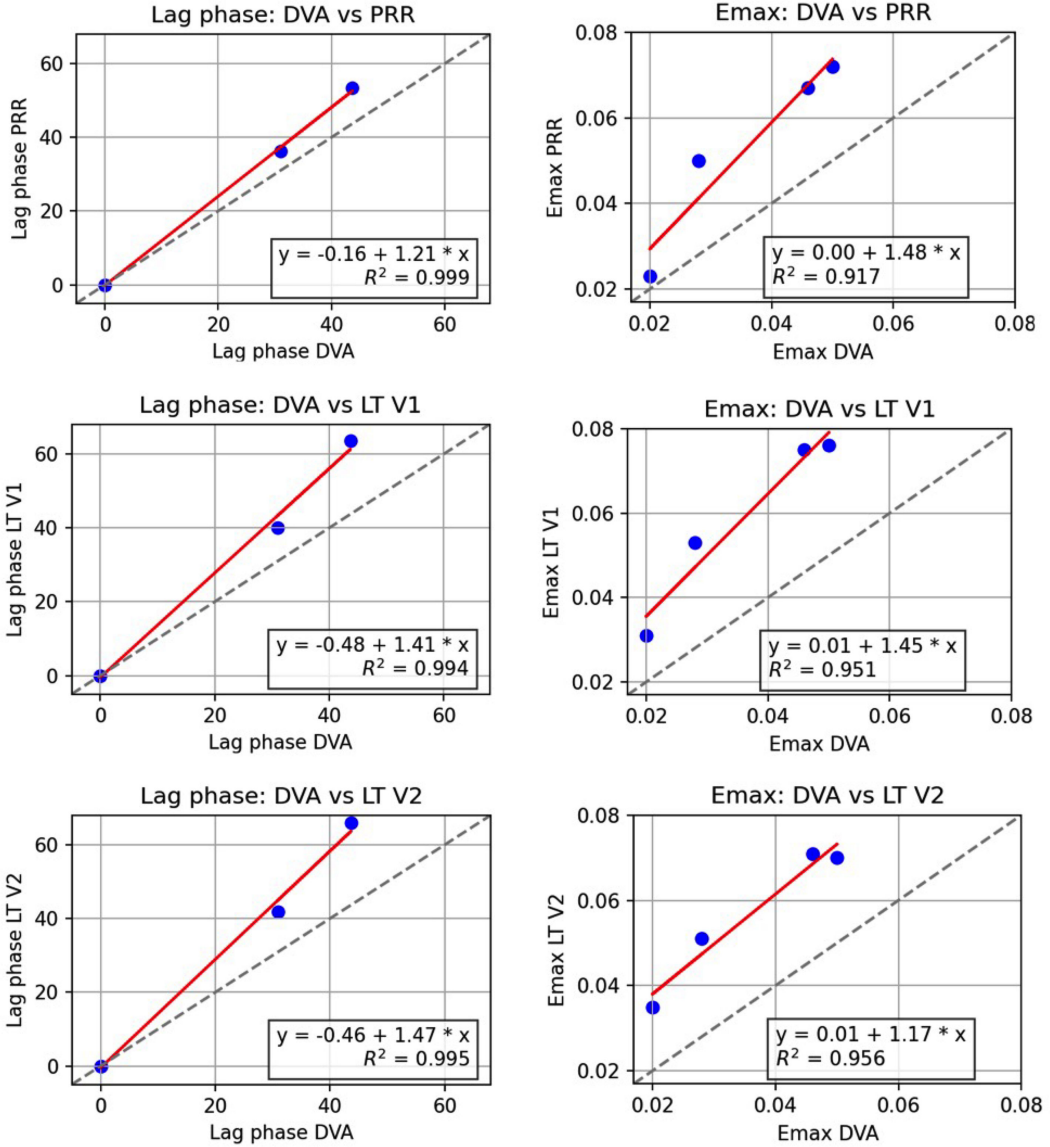
Comparison of PD parameter estimates from DVA and PRR. PD parameter (lag phase and *E*_max_) obtained from experimental DVA assays were compared to PD parameters from experimental and literature (LT V1 [8] and LT V2 [3]) PRR assays. The dashed, gray line indicates the identity of the two methods, the red line is the quadratic regression with the equation, and *R*^2^ value displayed in the plot.

We conducted a comparative linear regression analysis between DVA and the PRRs (our experimental [PRR] and LT version 1 & 2 [LT V1 and LT V2]), focusing on the estimated PD parameters for the four compounds, specifically lag phase and *E*_max_, as illustrated in Fig. 3 . This analysis aimed to identify linear relationships and estimation trends among the different methods. A strong qualitative agreement was observed between DVA and its PRRs, supported by high coefficients of determination (*R*²), indicating a solid quantitative correlation. However, despite these high correlations, the linear regressions revealed systematic differences in the parameter estimates (Fig. 3). Notably, DVA consistently displayed higher *E*_max_ values than the PRR assays, with slopes greater than 1 in all comparisons (1.48 for PRR, 1.45 for V1, and 1.17 for V2). This suggests that DVA tends to quantitatively estimate elevated maximal effects compared to our experimental PRR, the LT V1 PRR, and LT V2 PRR. Additionally, DVA also displays shorter lag phases compared to PRRs, with regression slopes ranging from 1.21 to 1.47. This indicates that DVA anticipates a shorter delay in reaching the observed effects compared to the other methods for slow-acting compounds.

A strong qualitative correlation was observed between our experimental PRR and LT V1 and V2 PRRs for both lag phase and Emax parameters (Fig. S1). However, LT V1 and V2 PRRs tended to quantitatively estimate slightly longer lag phase compared to our experimental PRR. Regarding *E*_max_, some differences emerged particularly with LT V2 estimating lower maximum effects. In contrast, our experimental PRR and LT V1 PRR exhibited better agreement for *E*_max_, with slopes close to 1.

### Rapid DVA assay accurately assesses drug sensitivity and resistance in immediate “ex vivo” *Plasmodium falciparum* field isolates

**Fig 4:**
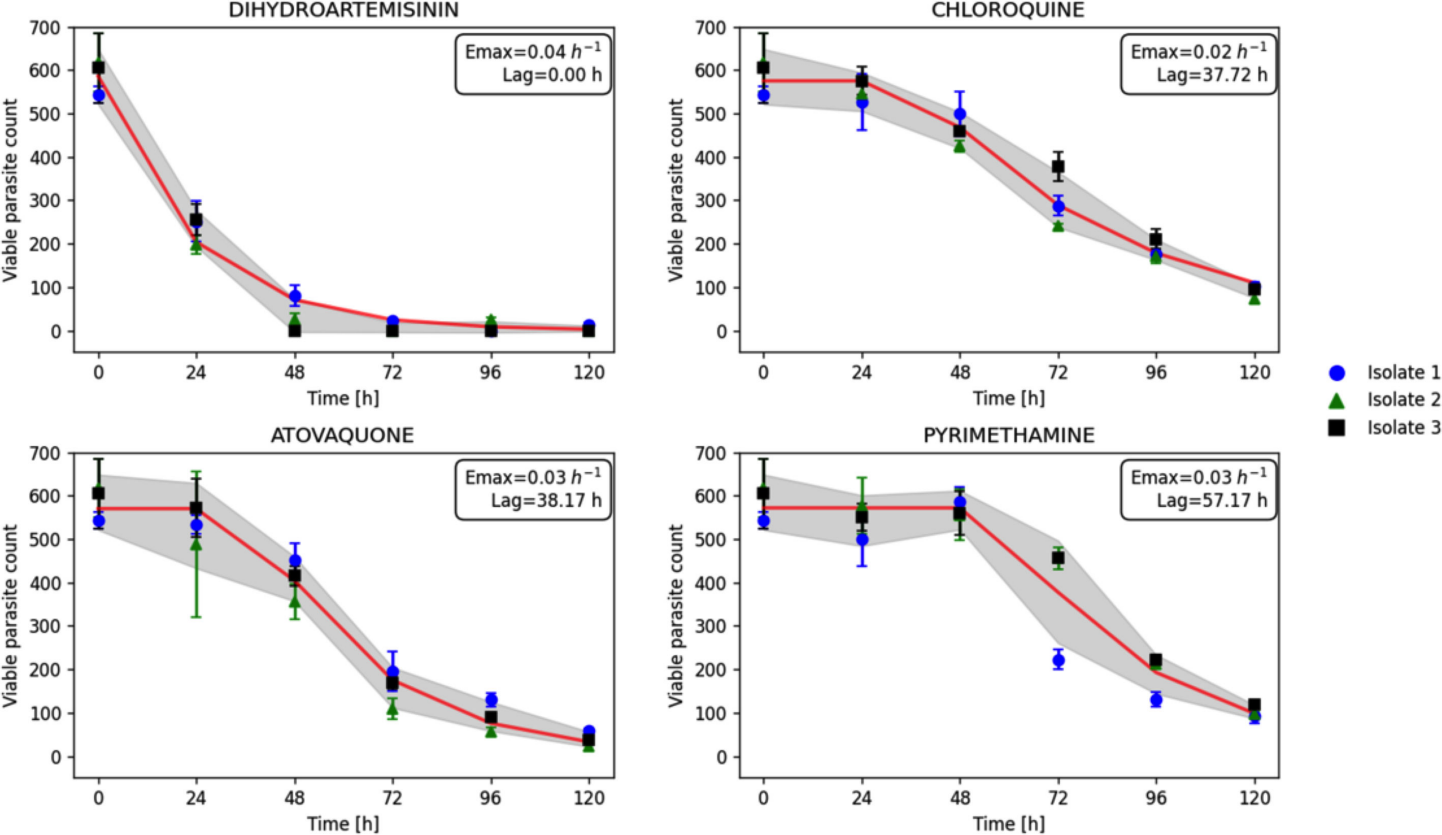
Applicability of DVA assay in clinical setting using IEV field isolates. The dots represent the observation data, while the line represents the model prediction. The maximum killing effect (*E*_max_) and lag phase (LAG) are estimated parameters (Table 2). Error bars represent SEM (each done in triplicate). The shaded area indicates the standard deviation.

To evaluate the robustness and applicability of the DVA assay, the efficacy of the four standard compounds (DHA, CQ, ATO, and PYRI) was evaluated on immediate “ex vivo” (IEV) *P. falciparum* field isolates (Fig. 4).

**TABLE 2:** Summary table of PD parameters estimates from the final DVA assay model: field isolates vs laboratory 3D7 strain parasites^*b*^

Drug	Parameters	Estimate (CI_95%_)	
		DVA (3D7)	DVA (field isolates)
Artemisinin	*E*_max_ [h^−1^]	0.050 [0.046–0.053]	0.044 [0.0394–0.0486]
	Lag phase [h]	0^*a*^	0^*a*^
Chloroquine	*E*_max_ [h^−1^]	0.046 [0.045–0.054]	0.0201 [0.0173–0.0229]
	Lag phase [h]	0^*a*^	37.7 [33.153–42.247]
Atovaquone	*E*_max_ [h^−1^]	0.02 [0.001–0.021]	0.0349 [0.0276–0.0422]
	Lag phase [h]	43.7 [41.4–46.6]	38.2 [34.045–42.355]
Pyrimethamine	*E*_max_ [h^−1^]	0.028 [0.022–0.037]	0.028 [0.0208–0.0352]
	Lag phase [h]	31.0 [24.3–37.7]	57.2 [50.516–63.884]

^*a*^ Fixed to final estimate. DVA, direct viability assessment. CI95%, 95% confidence intervals of the estimate.

^*b*^ DVA (field isolates) estimate parameters represent the mean of estimate parameters from the three field isolates.

The results demonstrated distinct patterns of parasite response among the three IEV field isolates. For all compounds, a reduction in parasite counts was observed with increasing exposure time, indicating time-dependent effects on viability. Notably, DHA exhibited the most rapid and consistent reduction in parasite counts across all isolates, characterized by minimal variability. This was reflected by consistently high *E*_max_ value 0.04 h^−1^ and low lag phase value 0, consistent with observations made using the *P. falciparum* 3D7 laboratory strain (Fig. 2).

Unlike DHA, which demonstrated consistent efficacy in both *P. falciparum* 3D7 and IEV field isolates, CQ shows significantly reduced efficacy, with *E*_max_ value 0.02 h^−1^ and higher lag phase value 37.7 h, suggesting a slower response in field isolate parasites. This delayed drug action was further investigated and corroborated by PCR analysis of field isolates DNA, which revealed the presence of the K76T mutation in the *Pfcrt* gene, know to confer CQ resistance, in all tested isolates (Fig. S2). This mutation was not present in the 3D7 laboratory strain.

ATO, while having a low Emax value of 0.03 h^−1^, demonstrated slightly faster killing in field isolates compared to the 3D7 strain. However, it remained a slow-acting compound in these field isolates as expected. The lag phase for ATO was consistent with its known pharmacodynamics, reflecting its delayed onset of action.

PYRI exhibited low a *E*max value of 0.02 h^−1^ and a higher lag phase of 57.17 h, indicating slower action in the field isolates. This reduced efficacy was further confirmed by PCR analysis, which identified mutations at position S108N in the *Pfdhfr* gene, known to confer PYRI resistance, in all tested isolates (Fig. S2). These mutations were not present in the 3D7 laboratory strain.

## DISCUSSION

Accurate evaluation of malaria parasite viability is crucial for determining the effectiveness of antimalarial drugs and supporting clinical development (4). This assessment is critical in the clinical setting, where the ability to rapidly determine parasite viability after treatment can directly influence therapeutic decisions and can help to ensure optimal outcomes for patients.

In this study, we aimed to evaluate the role of the DVA assay for assessing parasite viability by comparing its outcomes with the PRR assay. Our objective was to determine whether the DVA assay could serve as a reliable alternative, offering a faster and more practical method for evaluating parasite killing and, consequently, treatment efficacy. By aligning the outcomes of both assays, we provide a comprehensive comparison of the DVA, our experimental PRR, and the LT PRR assays. The use of modeling was instrumental to this study, given the distinctly different read outs of both assays, i.e., viable parasites for the DVA assay or viable parasite count for the PRR assay.

For the tested antimalarial drugs, the DVA and PRR assay were qualitatively and quantitatively in very good agreement and the pharmacologically distinct killing patterns ranging from fast to slow were well reproduced in the DVA assay. However, the modeling revealed a trend toward higher *E*_max_ and shorter lag phase for the DVA assay compared to the PRR assays.

A significant advantage of the DVA assay is its capability to directly measure viable parasites through flow cytometry (10). This method allows rapid assessment of parasite viability without the need for prolonged culture times, enabling real-time results. In contrast, the PRR assay involves a more complex process (i.e., labor-intensive and time-consuming), requiring continuous parasite culture for 14–21 days to allow parasite regrowth after drug removal. Additionally, the extended timeframe required for the PRR assay presents a significant limitation, particularly where cultivating field isolates is challenging. These isolates often experience high pressure as they adapt to *in vitro* conditions, potentially dying from adaptation stress rather than drug exposure. This complicates the accurate assessment of drug action.

Over the years, various technologies have been developed to assess parasite killing and treatment impact, each offering unique perspectives with distinct advantages and limitations. Microscopy provides valuable insights into *Plasmodium* developmental stages but lacks sensitivity and cannot reliably differentiate between live and dead parasites, potentially skewing drug efficacy assessments (3, 14). While polymerase chain reaction (PCR) assays offer high sensitivity for detecting parasitic DNA or RNA, they have notable limitations in assessing parasite viability. Quantifying viable parasites accurately with PCR is challenging because it measures genetic material rather than functional status, amplifying DNA from both live and dead parasites, potentially leading to misleading results about the true viability of the parasites (15, 16). This issue is also observed with DNA-based fluorochromes like Hoechst and SYBR Green I (13, 17), which can differentiate between *P. falciparum*-infected and uninfected red blood cells but cannot distinguish between live and dead parasites (18). Propidium iodide (PI), a fluorescent dye that does not permeate cell membranes and intercalates with DNA and RNA, is used to differentiate apoptotic and healthy cells based on membrane integrity. However, PI has limitations; studies have shown that in some actively growing cells, the cell walls can become permeable to PI, potentially leading to an underestimation of cell viability and inaccurate evaluations (19, 20). An alternative method for assessing parasite viability involves the use of radiolabeled hypoxanthine (21). This technique measures parasite proliferation by tracking the incorporation of hypoxanthine into the DNA and RNA of actively growing parasites. However, its accuracy can be compromised if hypoxanthine levels are insufficient or if parasites interact with certain antimalarial treatments, potentially leading to inaccurate results (3, 21). Furthermore, the use of radiolabeled hypoxanthine poses concerns about radiation exposure, waste management, and is impractical for field settings, requiring stringent safety measures and limiting its broader applicability. Similarly, the DVA assay also has limitations, including a higher quantification limit compared to the PRR, the need for access to a flow cytometer, and the reliance on a well-optimized gating strategy, which may present challenges in resource-limited environments.

In conclusion, although there are many techniques for assessing parasite viability, DVA and PRR stand out as prominent methods for assessing parasite viability, and our study shows that both yield similar results when quantifying the maximum possible killing rate and lag phase. While currently being the most widely used method, the PRR assay is complex and time-consuming, making it better suited for laboratory research than for clinical use, especially in resource-limited endemic areas (Figure S3). In contrast, DVA yields rapid results and is operationally simpler, and data remain accurate, making it a more effective choice for clinical settings that currently are basing its treatment decision on parasite clearance time (Fig. S3). Additionally, this approach not only will support the development of antimalarial therapies but could also enhance our understanding of the mechanisms underlying the mode of actions of these drugs, enabling better optimization of efficacy.

## MATERIALS AND METHODS

### Chemicals and materials

Standard anti-malarial drugs were purchased Thermo Scientific. Stock solutions were prepared in DMSO at 10 mM. The final DMSO concentration used in the experiments (<0.5%) had no inhibitory effect on parasite cultures.

### Parasite culture

*P. falciparum* field isolates were obtained from Faladié (Mali), while the reference 3D7 strain was provided by the USA National Institutes of Health (NIH). For *ex vivo* assays, freshly collected 3–6 h of venous blood containing *P. falciparum* isolates were processed. Blood samples (3–10 mL) were collected in acid-citrate-dextrose vacutainers (Becton-Dickinson, Franklin Lakes, NJ, USA) from infected individuals prior to antimalarial treatment, transported on ice packs (2–4°C) to the laboratory, and confirmed via Giemsa-stained thick/thin smears for quantification and speciation. These isolates, along with the reference 3D7 strain, were cultured *in vitro* using a standard protocol (22). Briefly, parasites were cultured in RPMI1640 medium supplemented with 25 mM HEPES, 0.5% (wt/vol) AlbuMAX I, 1.77 mM NaHCO_3_, 100 µM hypoxanthine, and 12.5 µg mL−1 gentamicin sulfate at 2% hematocrit under an atmosphere of 5% CO_2_, 5% O_2_, and 90% N_2_ at 37°C. Human O^+ve^ red blood cells (RBCs) were obtained from non-malaria-naive individuals through the Malian blood bank in Bamako (Mali).

### *In vitro* viability assay

Asynchronous cultures with a predominant ring population (ca. 70% rings) at 1% hematocrit and 0.5% parasitemia, distributed per well into 6-well plates, were incubated with the selected drugs (DHA, CQ, ATO, and PYRI) at concentrations corresponding to 10 times their respective IC_50_ values (Table 3).

**TABLE 3:** Antimalarial drug concentrations used to assess parasite viability

Drug	IC_50_ (nM)	10 × IC_50_ (nM)
DHA	1	10
CQ	24	240
ATO	1	10
PYRI	94	940

Parasites were sampled at various time points: untreated (0 h) and treated parasites (24 h, 48 h, 72 h, 96 h, and 120 h). For each time point, the compound was removed by washing three times with incomplete medium (RPMI1640 supplemented with 25 mM HEPES, 1.77 mM sodium bicarbonate, and 12.5 µg/mL gentamicin sulfate) before preparing the samples for the DVA and/or PRR assay. As a control, an untreated sample was included to monitor parasite growth over a period of 48 h. Compound and/or culture medium were replenished every 48 h.

### Assessment of parasite viability

#### Direct viability assessment assay

**Fig 5:**
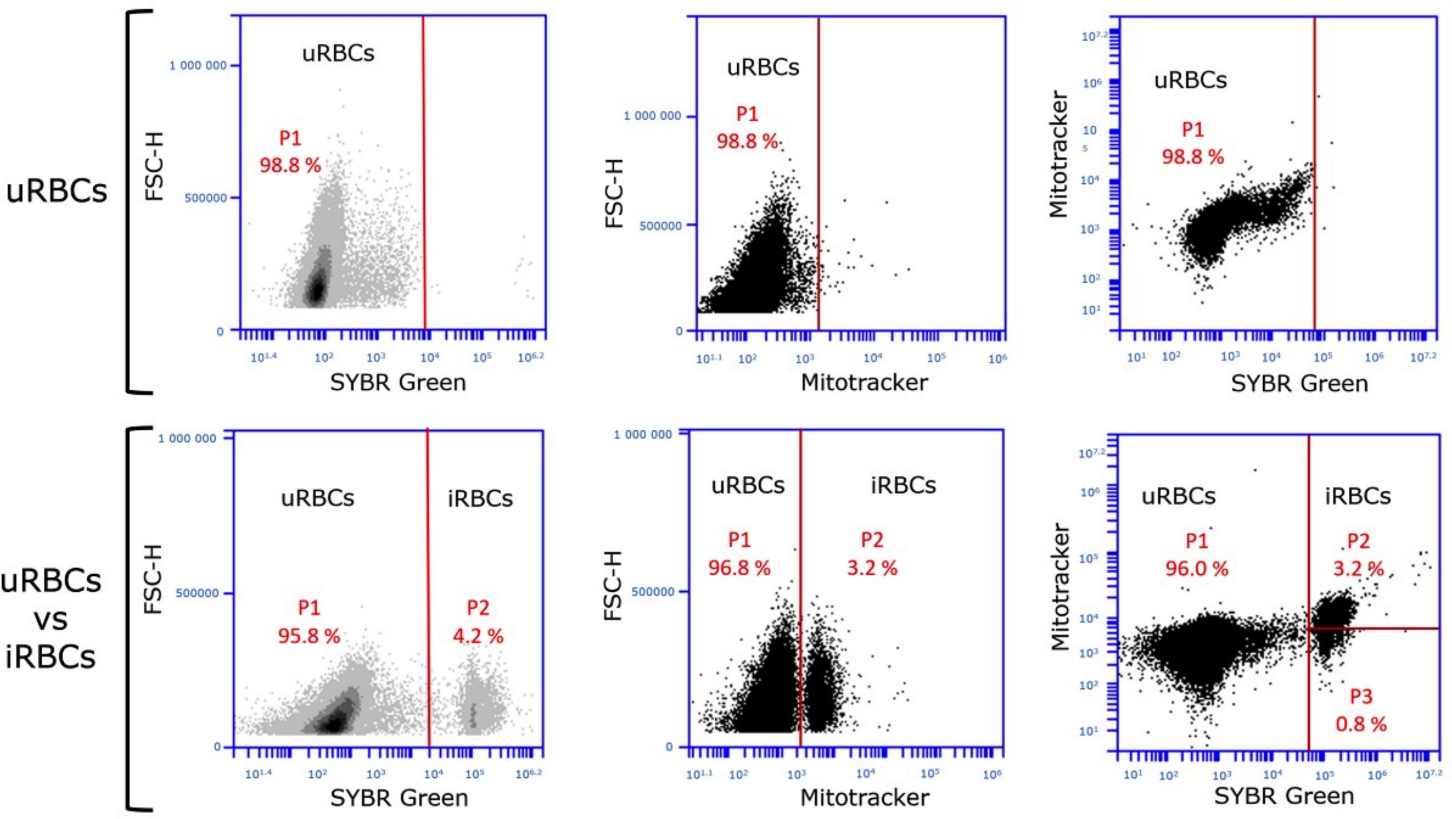
Optimizing gating strategies to identify infected red blood cells (iRBCs) and differentiate uninfected red blood cells (uRBCs) with SYBR Green and MitoTracker Staining. Each dot on the plot represents a single red blood cell, either infected or uninfected, that has passed through the cytometer laser. The combined data reveal two distinct populations: one representing uRBCs and the other representing iRBCs.

The washed parasites were resuspended in incomplete medium, divided into three technical replicates (eight for the 0 h time point), and then transferred to a 96-well plate. Parasite viability at different time points post-drug incubation was quantified by flow cytometry, as previously described (23). Briefly, infected RBCs (iRBCs) were stained with SYBR Green (ThermoFisher Scientific, Cat# M22426) and MitoTracker Deep Red FM (ThermoFisher Scientific, Cat# S7563) and then enumerated by flow cytometry (BD Accuri C6 Flow Cytometer). iRBCs, which contain parasite DNA and exhibit mitochondrial metabolic activity, were distinguished from uninfected RBCs (uRBCs) by their increased fluorescence. Gating was performed by plotting cells based on forward scatter (FSC) versus the fluorescence intensity of SYBR Green (FL1: 488 nm excitation/515–530 nm emission), MitoTracker (FL4: 644 nm excitation/665 nm long-pass emission), or a combination of both (FL4/FL1) to identify accurately the iRBC viable populations (Fig. 5). Combined gating using both dyes created a scatter plot to visualize and differentiate iRBCs from uRBCs based on their fluorescence (Fig. 4). Flow cytometry settings were kept consistent across all samples. The default FSC-H threshold of 80,000 was applied to exclude debris and small events, and the Gain Settings were maintained at the medium level for all fluorescence channels.

To establish the limit of quantification (LOQ), uninfected red blood cells (uRBC), which lack mitochondria, were included as a negative control. In parallel, *P. falciparum* 3D7 iRBCs at 1% parasitemia were serially diluted (1:3 ratio) across duplicate wells in 96-well plates to provide a reference range of mitochondrial signal intensity. The dilution point at which the MitoTracker signal from iRBCs matched the background signal of uRBCs was considered the LOQ (Fig. S4). The observed LOQ threshold corresponded to 46 viable parasites count for 0.08% parasitemia (approximately 60,000 total erythrocytes counted for detection) (Fig. S4). Parasitemia was expressed as the percentage of iRBCs with respect to the total uRBCs. For qualitative assessment, parasites were also visualized by microscopy.

#### Parasite reduction ratio assay

Three technical replicates of each washed aliquot (with eight replicates for the 0 h time point) were serially diluted (1/4) in 96-well plates (Sarstedt #83.3924) by adding fresh erythrocytes and new culture media. As previously described (3), parasites were cultured for up to 14 days to allow wells with viable parasites to develop detectable parasitemia. Medium was refreshed every 48 h, and fresh erythrocytes (up to 1.5% hematocrit) were added once a week to support parasite growth. At the end of the incubation period, culture media was removed, and the infected RBCs were stained with SYBR Green (ThermoFisher Scientific, Cat# M22426) and MitoTracker Deep Red FM (ThermoFisher Scientific, Cat# S7563). Additionally, for qualitative assessment, parasites were also visualized by microscopy.

For each technical replicate of a sample, the number of viable parasites was extrapolated using the following equation (equation 1):


(1) $$\text{ Pviable }=\left\{ \begin{array}{rl} X^{n-1},\text{ If }n & \geq 1\\ 0,\text{ If }n & =0 \end{array}\right.$$

where Pviable denotes the number of viable parasites, *X* represents the dilution factor applied during serial dilution, and *n* indicates the number of wells exhibiting detectable parasite growth. This information was then utilized to calculate the parasitemia specific to each well.

### Microscopy

The microscopic analysis of cultured parasites was performed on thin blood smears, fixed with 100% methanol and stained with Giemsa (Merck KGaA, Germany) at a dilution of 1:10 in distilled water. After incubation for 15–30 min at room temperature (25–27°C), the slides were rinsed with distilled water and dried at room temperature. Parasitemia was estimated by three independent microscopists by counting approximately 1,000 RBCs under oil immersion (×100) objective lens.

### Historic data for PRR assay

The data from the two PRR versions found in the literature were extracted by taking screenshots of the graphs from the respective source documents in PDF format and then saved as PNG files. Subsequently, the online tool **WebPlotDigitizer** (Rohatgi, A. (2023). *WebPlotDigitizer* (Version 5.2). Automeris (https://automeris.io) was used to import these images and define the *X* and *Y* axes by specifying the reference points and their respective values (*X*₁ = 0, *X*₂ = 120) and (*Y*₁ = 0, *Y*₂ = 5) for each image. The data points were then manually selected in three replicates. Once the extraction was completed, the data were exported as a CSV file to be used in the mathematical model. Importantly, all of the data used were derived from historical data sets (3, 8).

### Mathematical modeling of *in vitro* data

We developed a mathematical model using NONMEM software (version 7.5., ICON, Ellicot City, MD, USA) to estimate the maximum killing effect (*E*_max_) and the lag phase from the observed assay signal of the DVA assay or the parasitemia of the PRR assay.

The dynamics of the DVA assay signal or parasitemia concentration, termed *N*, is governed by the following differential equation:


(2) $$\frac{dN}{dt}=-E\times N$$

with


(3) $$E=\left\{ \begin{array}{ll} \text{ Emax }\times \text{ ONOFF } & \text{ IF }N>\text{ BSL }\\ 0 & \text{ ELSE. } \end{array}\right.$$

This mathematical model can quantify a delayed onset of the killing effect of drugs by incorporating a latency mechanism. The model begins by evaluating the time *T* against LAGTIME (lag phase). If *T* ≤ LAGTIME, the pharmacodynamic effect is inactive (ONOFF = 0). Beyond this time, the effect becomes active (ONOFF = 1). The effect *E* remains active until *N* reaches the baseline assay signal or parasitemia (BSL).

The ADVAN 13 subroutine (TOL = 9) of NONMEM was used for solving the differential equation. The LAPLACIAN algorithm was used for parameter estimation. The parameter estimates were extracted from converged models. 95% confidence intervals (CI 95%) were derived from the variance-covariance matrix of the final parameter estimates.

The estimated model parameters *E*_max_ and lag phase were compared across DVA, PRR, LT V1, and LT V2, and the correlation was assessed by using linear regression analysis in Python 3.12.4 software.

## Data Availability

All data and code used to reproduce the pharmacodynamic model simulations are available on GitHub at https://github.com/USTTB-MRTC/DVA_Data_Model.

## SUPPLEMENTAL MATERIAL

Supplemental material aac.01863-24-s0001.docx Fig. S1 to S4.

ASM does not own the copyrights to Supplemental Material that may be linked to, or accessed through, an article. The authors have granted ASM a non-exclusive, world-wide license to publish the Supplemental Material files. Please contact the corresponding author directly for reuse.
