# Supplementary material for: Clinically applicable parasite viability assay for rapid assessment of antimalarial pharmacodynamic endpoints
Source: Antimicrob Agents Chemother. 2025 Jun 17;69(8):e01863-24. doi: 10.1128/aac.01863-24 (PMC12326963; doi:10.1128/aac.01863-24)
Supplement: Supplemental material — Fig. S1 to S4. [file aac.01863-24-s0001.docx]

**Clinically applicable parasite viability assay for rapid assessment of antimalarial pharmacodynamic endpoints**

**Authors**

Mohamed Maiga^1^, Sebastian G. Wicha^2*^, Fatoumata Diallo^1^, Issa Traoré^1^, Abdoul Karim Samaké^1^, Fanta Sogore^1^, Ousmaila Diakité^1^, François Dao^1^, Djeneba Diallo^1^, Aliou Traoré^1^, Abdoulaye A Djimde^1^, Thomas Spangenberg^3^, Claudia Demarta-Gatsi^3*^, Laurent Dembele^1*^

**Affiliations**

^1^ Université des Sciences, des Techniques et des Technologies de Bamako (USTTB), Malaria Research and Training Centre (MRTC), Faculty of Pharmacy, Bamako, Mali

^2^ Department of Clinical Pharmacy, Institute of Pharmacy, University of Hamburg, Hamburg, Germany

^3^ Global Health R&D of the healthcare business of Merck KGaA, Darmstadt, Germany, Ares Trading S.A. (an affiliate of Merck KGaA, Darmstadt, Germany), Eysins, Switzerland

**Supplementary Material and Methods**

**Determination of half-maximal inhibitory concentration (IC_50_) values using *in vitro* culture of P. falciparum (3D7 strain))**

The half-maximal inhibitory concentrations (IC_50_) were determined through in vitro culture of *P. falciparum* 3D7 in complete RPMI-1640 medium, with a parasitemia of 0.5% and a hematocrit of 1%. Drug sensitivity to DHA, CQ, ATO, and PYRI was assessed using a combined Sybr Green assay, which measures parasite growth inhibition effect, and MitoTracker (MT), which evaluates parasite killing effect. Three-fold serial dilutions, of 10 000 nM, were tested for each compound in duplicated 96 well plate. After 48 hours of incubation at 37 °C in a 5% CO2 atmosphere, the plates were washed, stained, and then analyzed using an Accuri flow cytometer. IC_50_ values were calculated by nonlinear regression analysis using GraphPad Prism version 10, with data normalized to untreated controls.

**Parasitic gDNA extraction and sequencing - detection of *Pfcrt K76T and Pfdhfr S108N* mutations**

To identify mutations associated with chloroquine resistance (*Pfcrt* CQR) and *Pfdhfr*, we employed allele-specific restriction analysis (ASRA), which combines a two-step PCR with enzymatic digestion. DNA from reference strains, 3D7 (wild type) and Dd2 (mutant), provided by the Malaria Research Reagents Resource (MR4), served as positive controls to ensure the accuracy of the analyses. In addition to positive controls, we included negative controls (NC1 and NC2) that contained no DNA to verify the absence of contamination in the reactions. Enzymatic digestion was performed using the ApoI enzyme to detect the K76T mutation in *Pfcrt* and the AluI enzyme to identify the S108N mutation in *Pfdhfr*.

For the *Pfcrt* gene (K76T mutation), a two-step PCR was performed: the first amplification used primers CRT-P1 (CCGTTAATAATAAATACACGCAG) and CRT-P2 (CGGATGTTACAAAACTATAGTTACC) to produce a 537 bp fragment, followed by a second amplification with primers CRT-D1 (TGTGCTCATGTGTTTAAACTT) and CRT-D2 (CAAAACTATAGTTACCAATTTTG) to generate a 134 bp product (amplify a 134 bp segment around the 76 mutation). Enzymatic digestion with ApoI cleaved the wild-type (3D7) allele into 100 bp and 34 bp fragments, while the mutant (Dd2) allele remained undigested as a single 134 bp fragment.

For the *Pfdhfr* gene (S108N mutation), the first amplification with primers FR100-A (GGGGGGCAGTTACAACATATGTGA) and FR100-B (GGGGGCACATTCATATGTACTATTT) produced a 414 bp product, followed by a second amplification with primers FR108-D (CTAATTCTAAAAAATTACAAAATGT) and FR164-D3 (TTTCTTTTCTAAAAATTCTTGATAAACAACGGAACCTCTTA) to generate a 254 bp fragment (amplify a 254 bp segment around the 108 mutation). Enzymatic digestion with AluI cleaved the wild-type (3D7) allele into 176 bp and 78 bp fragments, whereas the mutant (Dd2) allele remained intact as a single 254 bp fragment. This methodology, as described in our previous works (1,2), allows for a clear distinction between sensitive and resistant strains while ensuring the reliability of results through the use of appropriate controls.

**Supplementary Figures**

**Figure S1: Comparison of PD parameter estimates from experimental and literature (V1 and V2) PRRs.**

PD parameters (lag phase and Emax) obtained from experimental PRR assay were compared to PD parameter from literature PRR assays, V1 (3) and V2 (4). The dashed, grey line indicates identity of the two methods, the red line is the linear regression with the equation and R^2^ value displayed in the plot.


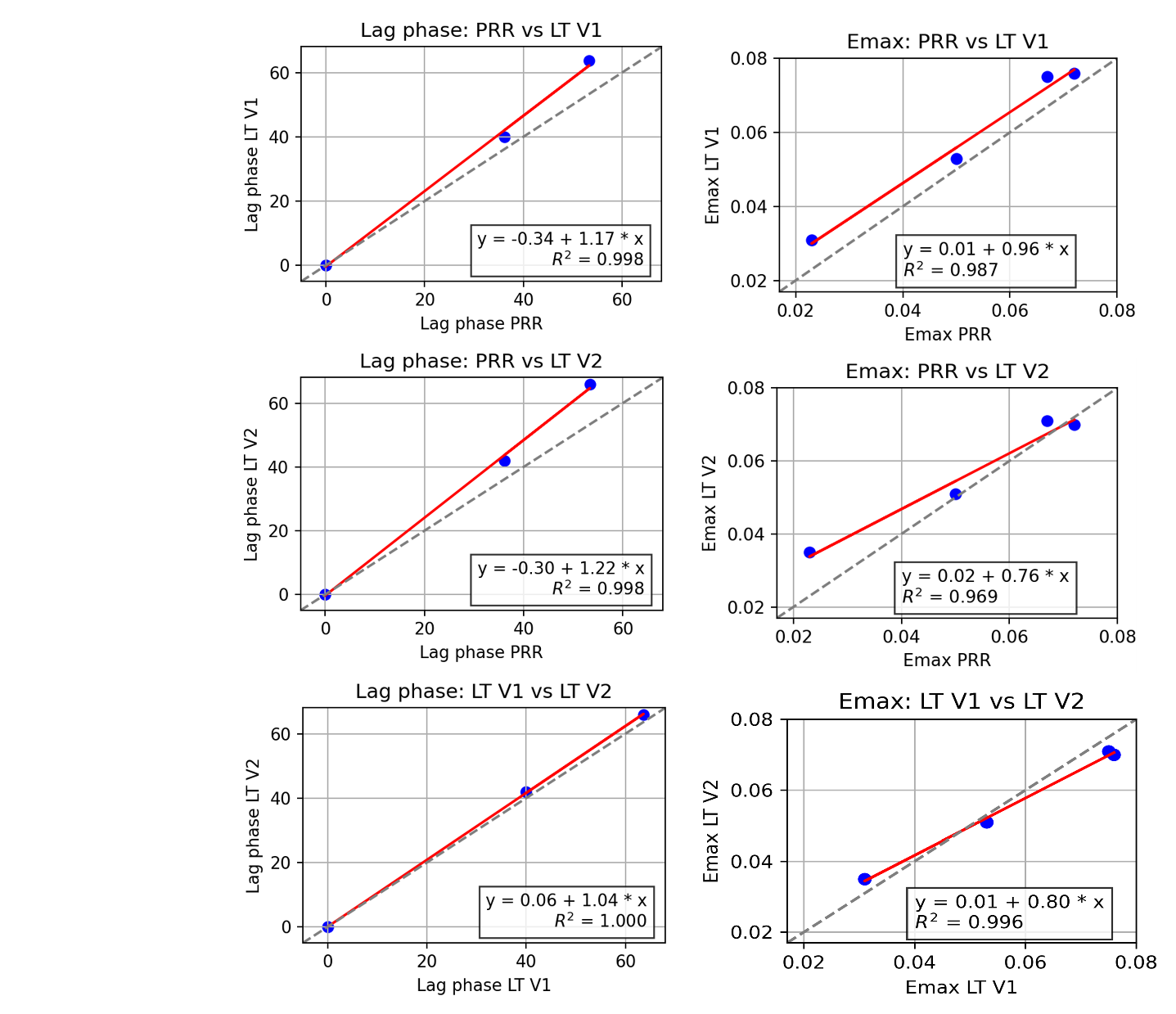


**Figure S2: Electrophoretic analysis of restriction fragments from Pfcrt and Pfdhfr genes in three immediate “Ex Vivo” (IEV) P. falciparum field isolates.** Bands on agarose gel against 100 bp DNA ladder. 3D7: control wild type (100 bp for *pfcrt*, 176 bp for *pfdhfr*); Dd2: control resistant to CQ and PYRI (134 bp for *pfcrt*, 254 bp for *pfdhfr*); isolate1, isolate2 and isolate3: three IEV *P. falciparum* field isolates; NC: Negative Controls.

**Figure S3: Schematic representation of the *ex vivo* direct viability assessment (DVA) and parasite reduction ratio (PRR) assays.** Intraerythrocytic *P. falciparum* 3D7 is cultured at 0.5% parasitemia and 2% hematocrit before being treated with the selected drugs (DHA, CQ, ATO, and PYRI) at concentration corresponding to 10 times their respective IC50 values. At designated time points, aliquots are extracted and washed. For the DVA assay, parasite viability is directly assessed using MT and SYBR Green staining through flow cytometry. In contrast, the PRR assay entails culturing parasites with fresh erythrocytes under limiting serial dilution conditions while monitoring parasite growth for up to 14 days before assessing parasite viability.


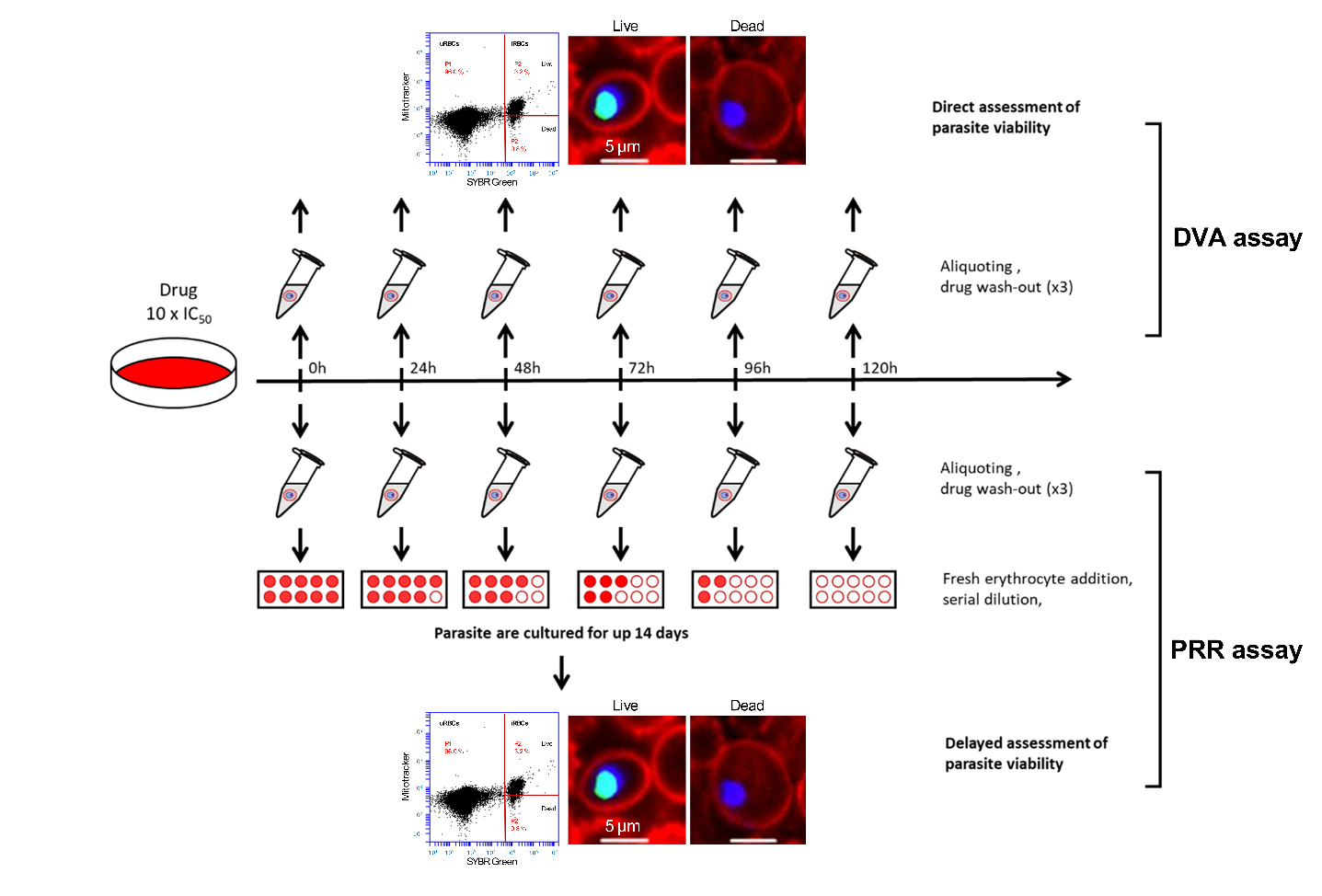


**Figure S4: Establishing the limit of quantification for the direct viability assessment (DVA) by MitoTracker (MT) staining**. Viable parasite count vs parasitemia. Red dash line: baseline, uRBCs. Green dash line: MT Limit of quantification (LOQ).


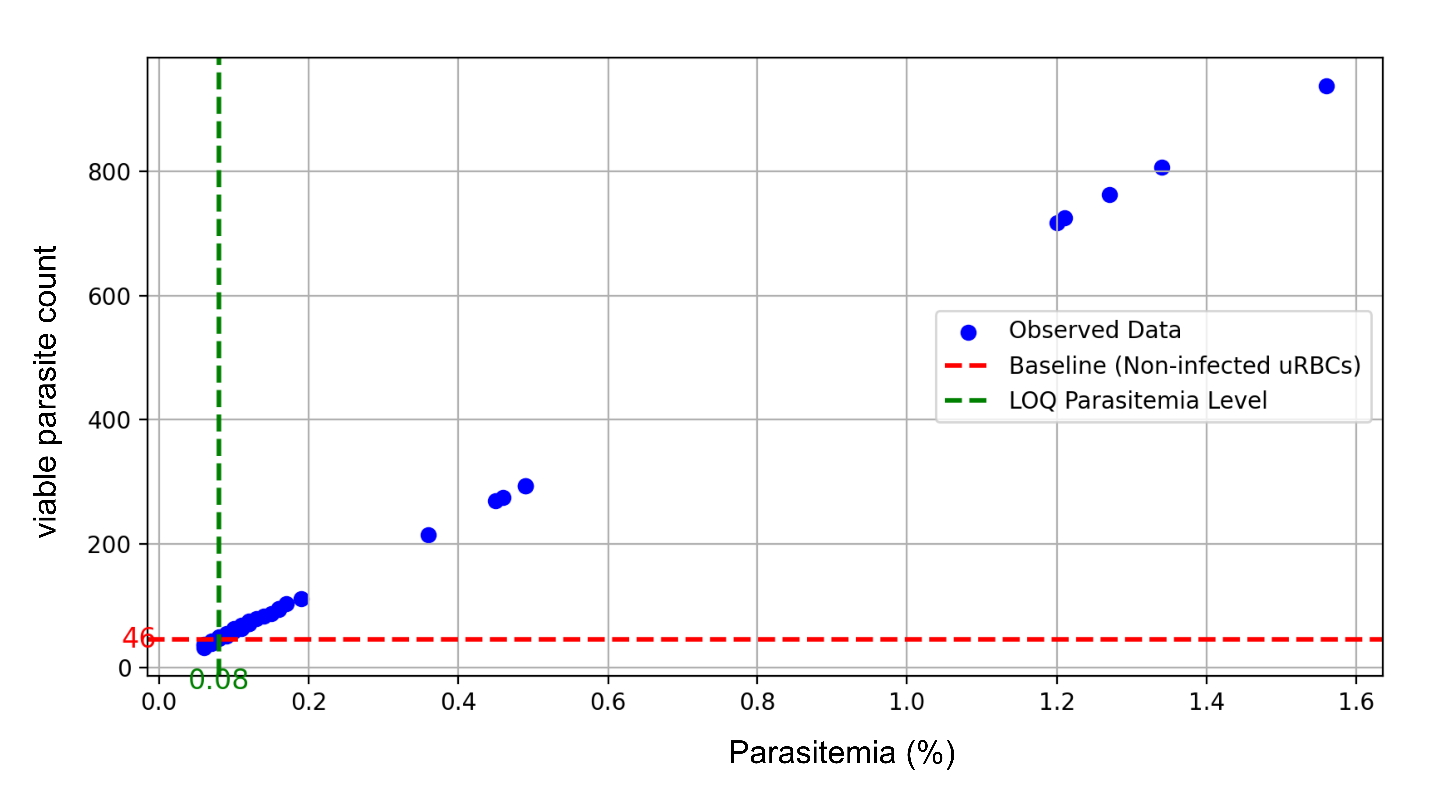


**References**

1. Maiga H, Lasry E, Diarra M, Sagara I, Bamadio A, Traore A, Coumare S, Bahonan S, Sangare B, Dicko Y, Diallo N, Tembely A, Traore D, Niangaly H, Dao F, Haidara A, Dicko A, Doumbo OK, Djimde AA. 2016. Seasonal Malaria Chemoprevention with Sulphadoxine-Pyrimethamine and Amodiaquine Selects Pfdhfr-dhps Quintuple Mutant Genotype in Mali. PLoS ONE 11:e0162718.

2. Djimde AA, Barger B, Kone A, Beavogui AH, Tekete M, Fofana B, Dara A, Maiga H, Dembele D, Toure S, Dama S, Ouologuem D, Sangare CPO, Dolo A, Sogoba N, Nimaga K, Kone Y, Doumbo OK. 2010. A molecular map of chloroquine resistance in Mali. FEMS Immunol Med Microbiol 58:113–118.

3. Sanz LM, Crespo B, De-Cózar C, Ding XC, Llergo JL, Burrows JN, García-Bustos JF, Gamo F-J. 2012. P. falciparum in vitro killing rates allow to discriminate between different antimalarial mode-of-action. PloS One 7:e30949.

4. Walz A, Duffey M, Aljayyoussi G, Sax S, Leroy D, Besson D, Burrows JN, Cherkaoui-Rbati MH, Gobeau N, Westwood M-A, Siethoff C, Gamo F-J, Mäser P, Wittlin S. 2023. The Parasite Reduction Ratio (PRR) Assay Version 2: Standardized Assessment of Plasmodium falciparum Viability after Antimalarial Treatment In Vitro. Pharm Basel Switz 16:163.
